# Lactation in horses

**DOI:** 10.1093/af/vfad003

**Published:** 2023-06-14

**Authors:** Amanda S Reiter, Sarah A Reed

**Affiliations:** Department of Animal Science, University of Connecticut, Storrs, CT 06269, USA; Department of Animal Science, University of Connecticut, Storrs, CT 06269, USA

**Keywords:** colostrum, horse, lactation, milk

**Implications**

- Mare colostrum contains immunoglobulin G and bioactive factors critical to the health of the newborn foal.
- Bioactive factors in mare’s milk can influence foal health and growth through protection from pathogens, establishment of the gut microbiota, and other growth supporting functions.
- Nutritive factors in mare’s milk support the continued growth and development of the foal through the first 6 to 9 mo of life.
- The structure and function of the mare’s udder contributes to low occurrence of mastitis and other diseases.

Lactation is the most energetically demanding phase of life for a mare. The foal relies entirely on the mare’s milk for approximately the first month of life, after which the foal begins to consume other food sources. Under human management, lactation in horses corresponds to a short period of time relative to gestation—mares lactate for 3 to 6 mo, or approximately 25 to 50% of their 11-mo gestation. In the wild, mares may lactate for over a year. Lactation is greatest 30 to 60 d after foaling, where daily milk production can range from 12 to 15 liters, representing consumption of 21 to 25% of the foal’s body weight (Morresey, 2012).

Postnatal development of the mammary glands begins during puberty, pauses, and is completed during the last 2 mo of gestation. The mammary tissue develops into lobes (groups) of alveoli, small sacs that synthesize milk (Figure 1). The alveoli drain into a duct system, which collects the milk and provides a route for the milk to exit the udder during suckling. Unique to the mare, each half of her udder contains two separate mammary gland complexes, which drain into separate teat cisterns and distinct teat canals. The size of the cisterns in the mare are similar to that of sheep and goats, but smaller than cows (Dzidic et al., 2002; Dzidic, 2003). Each of her two teats has two separate openings, thus, the milk produced in each mammary gland remains separate until it reaches the foal’s mouth. This is distinct from the cow, ewe, and goat, where milk from have a single teat canal and orifice.

**Figure 1.**
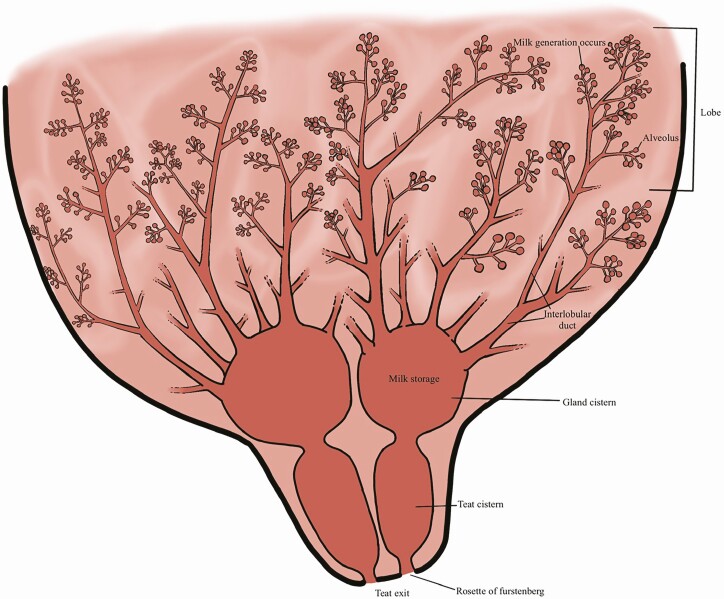
Equine udder anatomy. Milk generation occurs in the alveoli, and milk is “moved” through interlobular ducts into the gland and teat cisterns. Each side of the udder has two mammary glands, which drain into separate teat cisterns and out of the teat through the rosette of furstenberg. Photo credit: Daniela Johnson, University of Connecticut.

The colostrum and milk produced by the mare are critical for survival of the foal. Colostrum is the first milk secretion following parturition and is vital to the foal’s early growth, development, and immune function. It is rich in nutrients, providing the foal with essential amino acids, bioactive proteins, immunological factors, and antioxidants. Bioactive components of colostrum include lipases and proteinases to aid in the digestion of fats and proteins, respectively, and antioxidants like catalase and peroxidases that protect the neonate against oxidation of proteins, lipids, and DNA. However, the most widely studied component of colostrum are the immunoglobulins.

Colostrum is critical for the transfer of passive immunity and proper development of the immune system in the foal. At birth, the foal’s immune system is not mature, so colostral immunoglobulins are necessary to protect the neonate from environmental pathogens and support development of the immune system. Immunoglobulins account for approximately 60% of the protein in colostrum. Immunoglobulin G (IgG) is the predominant immunoglobulin in equine colostrum, with IgA occurring at a lower concentration. Cattle and other ungulates have a similar IgG/IgA colostrum profile, however, in humans, IgA is predominant with little IgG present. Unlike humans, the structure of the equine placenta limits its permeability to immune modulating proteins like immunoglobulins. Thus, unlike babies which are born with circulating IgG concentrations similar to their mothers because these pass through the human placenta (reviewed in Palmeira et al., 2012), foals are born with no immunoglobulins present in circulation and limited ability to resist disease. Since the transfer of immunoglobulins to foals does not occur in utero, immunoglobulins must be transferred through colostrum. In horses, the transfer of IgG from maternal circulation to the accumulating colostrum begins 2 wk before parturition (Peaker et al., 1979). Colostrum immunoglobulin concentrations decrease around 12 h postpartum (Pasquini et al., 2005), and remains low for the remainder of lactation, which is likely an adaptation as the foal is only able to absorb the immunoglobulins for the first 6 to 12 h of life.

The transition from colostrum to milk occurs within 2 d following parturition; however, the complete transition to mature milk occurs gradually over several weeks. The composition of mature milk supports continued growth of the foal and includes nutritive factors (those that are used directly as nutrition) and bioactive factors (those that have non-nutritive functions).

Equine milk has a low energy content relative to bovine and human milk (Table 1), which is due to correspondingly limited fat and carbohydrate content. Equine milk contains slightly less lactose than human milk, and bovine milk has less lactose than both (Uniacke-Lowe et al., 2010). However, due to the frequent suckling, foals receive over half of their daily calories from lactose (Martin et al., 1992).

**Table 1.** Composition of equine, bovine, and human colostrum, and mature milk

Item^1^	Equine^2^	Bovine^2^	Human^4^
Colostrum			
Protein, g kg^−1^	128.9 (63–191)	99.6 (33–135.8)	20.0 (12–36)
Immunoglobulins, g kg^−1^	79.2 (49–107)	52.3 (31–87)	18.79
Fat, g kg^−1^	14.5 (6.9–29)	46.3 (36–65)	34.3 (28–38)
Lactose, g kg^−1^	39.5 (33–46)	34.7 (25–48)	63.0 (51–70)
Milk			
Gross energy, kJ kg^−1^	2008	2820	2832.5
Protein, g kg^−1^	21.5 (15–28)	32.5 (31–38)	15.2 (9–21.4)
NPN × 6.38, %	11.21	5.23	20.42
Casein, %	50.00	77.23	26.06
Whey protein, %	38.79	17.54	53.52
Casein: Whey Protein	1.1:1	4.7:1	0.4:1
b-Lactoglobulin, %	30.75	20.1	Absent
α-Lactalbumin, %	28.55	53.59	42.37
Immunoglobulins, %	19.77	11.73	18.15
Serum albumin, %	4.45	6.2	7.56
Lysozyme, %	6.59	Trace	1.66
Lactoferrin, %	9.89	8.38	30.26
Fat, g kg^−1^	12.1 (5–20)	36.1 (35–9)	36.4 (35–40)
Lactose, g kg^−1^	63.7 (58–70)	48.8 (44–49)	67 (63–70)
Ash, g kg^−1^	4.2 (3–5)	7.8 (7–8)	2.2 (2–3)

^1^Data are presented as averages, with ranges in parentheses.

^2^ Malacarne et al., 2002; Pasquini et al., 2005; Ciesla et al., 2009; Csapo et al., 2009; Uniacke-Lowe et al., 2010; Pecka et al., 2012; Salimei and Fantuz, 2012; Markiewicz-Keszycka et al., 2015; Pieszka et al., 2016

^3^ Foley et al., 1978; Malacarne et al., 2002; Kehoe et al., 2007; Godhia and Patel, 2013; Puppel et al., 2019; Playford and Weiser, 2021; Polidori et al., 2022

^4^ Malacarne et al., 2002; Stelwagen et al., 2009; de Halleux and Rigo, 2013; Godhia and Patel, 2013; Polidori et al., 2022

Further contributing to the low energy content, equine milk has a low fat content compared with bovine and human milk (Uniacke-Lowe et al., 2010). Equine milk contains less triglycerides than human and bovine milk but is rich in free fatty acids. Medium chain fatty acids are predominant in equine milk and are generally absorbed as free fatty acids. Long chain fatty acids are predominant in human milk and short chain fatty acids are predominant in bovine milk (Pieszka et al., 2016). Equine milk has a greater concentration of polyunsaturated fatty acids compared with both bovine milk and human milk(Pieszka et al., 2016), which is likely a combination of dietary forage intake and the limited hydrogenation of fatty acids by gut microbes that occurs before absorption.

Mature equine milk has more crude protein than human milk but less than bovine milk (Table 1). The essential amino acid composition in equine milk is similar to that of bovine and human milk; however, while equine milk contains more essential amino acids compared with cows’ milk, human milk contains 10× greater amounts of essential amino acids than equine. Interestingly, concentrations of taurine, an essential amino acid necessary for many cellular processes, are greater in equine milk than bovine milk but only one tenth of the concentrations in human milk (Uniacke-Lowe et al., 2010).

An important characteristic of equine milk is the whey to casein protein ratio. Whey proteins are more readily digestible compared with casein proteins contributing to the greater digestibility of equine (1.1:1) and human (0.4:1) milk compared with bovine milk (4.7:1; Table 1). The whey protein fraction of milk consists of β-lactoglobulin, α-lactalbumin, immunoglobulins, lysozyme, and lactoferrin. The casein fraction consists of α-caseins, β-casein, κ-casein, and γ-casein. β-casein is the predominant casein found in equine and human milk where α-caseins are predominant in bovine milk. Casein protein forms micelles that transport important amino acids and macro-elements like calcium and phosphorus (Uniacke-Lowe et al., 2010). β-lactoglobulin and α-lactalbumin are the majority of whey proteins in mature equine milk. β-lactoglobulin is present in both equine and bovine milk but absent in human milk and is one of the primary allergenic proteins in milk. The function of β-lactoglobulin is not well defined; however, it is proposed that β-lactoglobulin binds vitamin A transporting it through to the small intestine for absorption (Perez and Calvo, 1995). In contrast, equine, bovine, and human milk all contain α-lactalbumin. Equine and human milk have similar concentrations of α-lactalbumin, and both are greater than bovine milk. α-lactalbumin is involved in the conversion of glucose and galactose to lactose, an important carbohydrate source for the foal (Uniacke-Lowe et al., 2010).

Immunoglobulins are also present in mature milk, but in contrast to colostrum, IgA is the principal immunoglobulin. This is similar to human immunoglobulin content in mature milk; however, in bovine milk, IgG is still the predominant immunoglobulin. Equine milk also contains other proteins to protect the foal from pathogens such as lysozyme and lactoferrin. Lysozyme has antimicrobial properties that can help protect the foal from bacterial infection and may aid in the development of the gut microbiota. Equine milk contains more lysozyme than human and bovine milk (Uniacke-Lowe et al., 2010). Lactoferrin also possesses antimicrobial properties by inhibiting bacterial growth. Especially in young animals, lactoferrin can improve nutrient digestion through increasing the mucosal surface of the intestine (Superti, 2020). Lactoferrin may also promote establishment of the gut microbiota in the newborn, particularly promoting establishment of “healthy” bacteria including lactobacillus and bifidobacterium. Equine milk has 18% greater concentrations of lactoferrin compared with bovine but 67% less than human milk (Uniacke-Lowe et al., 2010). The different concentrations of lactoferrin lysozyme may support the development of the gut microbiota unique to each species, in addition to providing protection from pathogens.

Mature equine milk contains vitamins and macro-elements. However, the concentrations of these are highly dependent on the mare’s diet and stage of lactation. Generally, B vitamins and vitamin A occur in similar concentrations in equine, bovine, and human milk. Mare milk has similar concentrations of vitamin D_3_ compared with bovine milk but 41× greater concentration than human milk (Pieszka et al., 2016). Equine milk contains 72% greater concentration of vitamin C compared with bovine milk but 19% less than human milk. The concentration of vitamin K_2_, essential for bone development, is similar to bovine milk but nearly 18x greater than human milk. In general, concentrations of macro-elements in equine milk are greater than in human milk and less than in bovine milk. However, there are large differences in the calcium and phosphorus concentrations. Equine milk has 21% less calcium and 20% less phosphorus than bovine milk, but equine milk contains 66% more calcium and 60% more phosphorus than human milk. However, the calcium to phosphorus ratio is similar in equine (1.3:1) and bovine milk (1.3:1) while human milk has a lower ratio (0.92:1).

The mare experiences relatively low frequency of mastitis, agalactia, and other mammary pathologies (reviewed in Hughes, 2021). Agalactia, or absence of milk production, can be temporary or “permanent” in mares. The most common cause of agalactia in the United States is due to fescue toxicosis. Mares grazing endophyte infected fescue pastures have limited mammary development and produce little to no milk. Mastitis is infection of the mammary gland. The relatively small capacity of the udder (in comparison with the dairy cow), frequent emptying as the foal nurses, and management of lactating mares likely contribute to the low frequency of mastitis. In particular, management practices such as providing foals with creep feed which leads to less suckling and reducing mare feed intake during the drying off period are likely key to helping prevent mastitis. The mare also has a relatively low frequency of neoplasias, or mammary tumors. However, mares with mammary tumors generally have a poor prognosis, as they are generally malignant.

The unique structure and function of the mare’s udder contribute to the successful production of colostrum and milk, which are critical for the health and development of the foal, and low frequency of mastitis and other mammary diseases.

## About the Authors

Amanda S. Reiter MS

Amanda Reiter is a graduate research assistant in the Department of Animal Science at the University of Connecticut. Amanda earned her B.S and M.S. in Animal Science at the University of Minnesota with an interest in animal nutrition and physiology. As a PhD candidate working in the Fetal Programming Research Group, her research focuses on evaluating how maternal nutrition during gestation influences offspring inflammation and oxidative stress, specifically the response in skeletal muscle. In addition, Amanda has an interest in how inflammation and oxidative stress impact skeletal muscle growth and development particularly how these factors alter muscle satellite cell function.

Sarah Reed, PhD

Sarah Reed is an Associate Professor in the Department of Animal Science at the University of Connecticut. Dr. Reed earned her M.S. and Ph.D. in Animal Science and then spent two years as a post-doctoral research associate in the Department of Physical Therapy at the University of Florida. Her current research interests include the impacts of maternal diet during gestation on offspring growth and development, and the effects of exercise training and detraining in horses. Dr. Reed enjoys teaching, in particular introducing undergraduate students to research through class and research opportunities.
